# Enhancing Diagnosis in Squamous Cell Carcinoma: Non-Invasive Imaging and Multimodal Approach

**DOI:** 10.3390/diagnostics15081018

**Published:** 2025-04-16

**Authors:** Mircea Negrutiu, Sorina Danescu, Monica Focsan, Stefan Cristian Vesa, Adelina Cadar, Stefan Vaida, Alexandra Oiegar, Adrian Baican

**Affiliations:** 1 Department of Dermatology, “Iuliu Hatieganu” University of Medicine and Pharmacy, 400012 Cluj-Napoca, Romania; negrutiu.mircea.ionut@elearn.umfcluj.ro (M.N.); adrian.baican@umfcluj.ro (A.B.); 2 Nanobiophotonics and Laser Microspectroscopy Center, Interdisciplinary Research Institute on Bio-Nano-Sciences, Babes-Bolyai University, 400084 Cluj-Napoca, Romania; monica.iosin@ubbcluj.ro; 3 Department of Functional Sciences, Discipline of Pharmacology, Toxicology and Clinical Pharmacology, Faculty of Medicine, “Iuliu Haţieganu” University of Medicine and Pharmacy, 400012 Cluj-Napoca, Romania; stefanvesa@gmail.com; 4 Faculty of Medicine, “Iuliu Hațieganu” University of Medicine and Pharmacy, 400012 Cluj-Napoca, Romania; cadaradelina99@gmail.com; 5 Department of Plastic Surgery, “Iuliu Hațieganu” University of Medicine and Pharmacy, 400012 Cluj-Napoca, Romania; dr.stefanvaida@gmail.com; 6 Municipal Clinical Emergency Hospital, 300041 Timișoara, Romania; alexandra.oiegar@gmail.com

**Keywords:** cutaneous squamous cell carcinoma, dermoscopy, ultrasonography, ex vivo confocal microscopy, non-invasive imaging

## Abstract

**Background/Objectives**: Cutaneous squamous cell carcinoma (cSCC) is the second most common skin cancer, with diverse clinical presentations. This study aims to correlate findings from dermoscopy, ultrasonography, ex vivo confocal microscopy, and histology to improve diagnostic accuracy and guide better clinical management of cSCC. **Methods**: This cross-sectional study, conducted between July 2022 and December 2024, included 26 patients with 35 clinically suspicious cSCC tumors, analyzed through clinical, dermoscopic, high-frequency ultrasound (HFUS), ex vivo confocal fluorescence microscopy (FCM), and histopathology. Tumors were evaluated for various clinical, imaging, and histopathological criteria, such as tumor thickness, vascularization, differentiation degree, and invasion level, with FCM applied to 24 tumors for advanced microscopic analysis. **Results**: The study analyzed 35 cases of histopathologically confirmed cSCC, finding that invasive SCC was associated with greater tumor thickness, increased vascularization, and ulceration on both ultrasound and dermatoscopy, while in situ SCC showed homogeneous echogenicity and specific dermoscopic patterns like dotted vessels and white halos. Strong correlations were identified between ultrasound and histopathological measurements of tumor thickness and invasion depth, and confocal microscopy revealed that features like plump bright cells and nest-like structures were linked to invasive and poorly differentiated tumors. **Conclusions**: This study uniquely integrates advanced imaging techniques—dermatoscopy, skin ultrasound, and ex vivo confocal microscopy—with histopathological analysis to provide new insights into tumor grade, vascularity, and invasion depth in cSCC, enhancing non-invasive diagnosis.

## 1. Introduction

Cutaneous squamous cell carcinoma (cSCC) is a prevalent skin cancer marked by the malignant proliferation of epidermal keratinocytes and is categorized as a keratinocyte carcinoma alongside basal cell carcinoma (BCC). It manifests in two forms: in situ (Bowen’s disease) and invasive [1].

Based on disease progression, cSCC is classified into common primary and advanced forms. The common primary type, which is the most frequent, includes non-metastatic lesions that are generally treatable. These can be further divided into low-risk and high-risk cSCC, with high-risk cases exhibiting features that increase the likelihood of recurrence and metastasis but remain treatable with surgery or radiotherapy. Advanced cSCC is further distinguished as locally advanced (lacSCC) or metastatic (mcSCC), which includes both locoregional and distant metastases [2].

As the second most common type of skin cancer, cSCC accounts for approximately 20% of keratinocyte carcinomas [3]. The ratio of BCC to cSCC varies between 2:1 and 4:1 [4].

Global incidence data indicate that cSCC rates increase with age, male sex, and lower latitudes [2]. The occurrence of multiple primary tumors in a single patient is strongly associated with aging [5]. In the UK (2013–2015), 62.7% of cSCC cases occurred in men, with a median age of 80 years and an annual incidence rise of 5% [6,7]. In Norway, age-adjusted rates surged nine-fold in females and six-fold in males (1963–2011), particularly in those aged 70–79 [8]. The USA reported a 263% increase (1976–2010), with a notable rise in women and individuals under 40 [9].

cSCC presents with diverse clinical appearances influenced by factors such as tumor size, differentiation, pigmentation, location, and skin type. It predominantly develops on sun-exposed areas, including the head, neck, forearms, and hands. The presence of multiple actinic keratoses (AK) is a well-established risk factor for cSCC development in individuals without a prior history of the disease [10,11].

In its early, minimally invasive stage, cSCC typically appears as a small, flesh-colored papule or plaque with a scaly or hyperkeratotic surface, often resembling hyperplastic AK or in situ SCC (Bowen’s disease). As it progresses, the lesion enlarges at a variable rate, frequently developing ulceration, crusting, and induration upon palpation. In some cases, cSCC may be pigmented. The degree of differentiation also influences the clinical presentation. Well-differentiated cSCC often appears as a hyperkeratotic, verrucous tumor with a crateriform structure, while poorly differentiated cSCC tends to present as a red, non-keratotic mass that is frequently ulcerated or prone to bleeding [2].

Dermoscopy plays a vital role in the clinical assessment of skin tumors, particularly cSCC. The dermatoscopic features of cSCC vary depending on the histopathological grade of differentiation. However, while dermoscopy is widely used for evaluating suspicious lesions, its ability to distinguish AK from early-stage cSCC remains limited, especially in detecting subclinical lesions. Large-scale studies assessing its diagnostic accuracy are still needed [12].

Beyond dermoscopy, other non-invasive imaging techniques, such as high-frequency ultrasound (HFUS), reflectance confocal microscopy (RCM), line-field confocal optical coherence tomography (LC-OCT), and optical coherence tomography (OCT), are emerging as valuable tools for skin cancer diagnosis [13]. HFUS provides essential information on tumor size, thickness, vascularization, and local invasion, aiding in both diagnosis and surgical planning. Studies have shown its utility in differentiating high-risk BCC from SCC based on echogenicity and vascular patterns [14].

RCM, though capable of identifying features with strong histopathological correlation (such as parakeratosis, atypical keratinocytes, and vascular alterations), is limited by its shallow laser penetration, making full-thickness tumor evaluation difficult. While not yet recommended for routine clinical use, RCM may have a role in distinguishing cSCC from BCC [15,16,17].

Similarly, LC-OCT and OCT offer deeper vertical imaging and could be particularly useful in differentiating in situ from early invasive cSCC [18,19,20].

Despite advancements in non-invasive imaging, biopsy and histopathological examination remain the gold standard for skin cancer diagnosis [2]. However, considering the impracticality of multiple biopsies in cases of widespread AK, non-invasive imaging techniques are becoming increasingly relevant in guiding biopsy decisions and improving diagnostic accuracy.

The primary objective of this study was to establish a meaningful correlation between the diagnostic criteria outlined in various investigative techniques, including dermatoscopy, ultrasonography, ex vivo confocal microscopy, and histology, specifically for cSCC. This correlation analysis focused on the most prevalent cSCC subtypes, aiming to enhance understanding of how these distinct diagnostic methods align in assessing and characterizing the disease. By comparing the diagnostic criteria across these modalities, the study sought to improve the accuracy of cSCC diagnosis, providing valuable insights into its clinical evaluation and management.

## 2. Materials and Methods

We conducted a cross-sectional study between July 2022 and December 2024 on patients with highly suspicious lesions of cSCC (a tumor exhibiting the following findings: persistent scaly or crusted plaques, nodular growth, ulceration or non-healing sores, and an erythematous or hyperkeratotic appearance), from the dermatology department of our institution. From the total of 60 patients selected for the study, after applying the inclusion criteria (clinically suspicious lesions of in situ or invasive squamous carcinoma, tumors to be excised) and the exclusion criteria (negative histopathological result for cSCC, patients who did not present themselves for excision or did not perform imaging explorations), a total of 26 patients remained, from whom 35 tumors were analyzed.

The study received approval from the Ethics Committee at both the University and the local hospital, under protocol ID number DEP 154/9, dated May 2022. Prior to participation, all patients were given a detailed explanation of the study protocol and eligibility criteria. Written consent was obtained from each participant before their inclusion. The study adhered to the principles outlined in the Declaration of Helsinki for medical research.

Demographic data and medical history were retrieved from each patient. During the dermatological clinical examination, parameters such as age, sex, phototype, tumor location and delimitation, clinical diameter, history of cSCC, and presence of ulceration and keratin crusts on the surface were monitored. Immunosuppression was also recorded as a potential high-risk factor for cSCC in our study. However, we found that none of our patients were immunosuppressed at the time of data collection. This factor did not influence the clinical presentation of cSCC in our cohort.

Subsequently, the tumors were analyzed using non-invasive imaging techniques (dermatoscopy, videodermatoscopy, and high-frequency ultrasound (HFUS)). The tumors were excised with oncological safety margins according to the hospital protocol and analyzed histopathologically. A total of 24 tumors were studied using an ex vivo confocal fluorescence microscope (FCM).

### 2.1. Ultrasound Acquisition

Ultrasonographic images were obtained using high-frequency ultrasound (HFUS) (Philips Affiniti (Philips Healthcare, Amsterdam, The Netherlands)) by two experienced dermatologists. The HFUS system was fitted with a high-resolution linear transducer (20 MHz), enabling detailed examination of the epidermis, dermis, hypodermis, and deeper structures, including the muscle fascia. Tumors were assessed in both thyroid and cutaneous modes, and B-mode together with color Doppler imaging were conducted for all lesions.

By applying the linear transducer perpendicularly and gently to the surface of the tumor, in a sufficient amount of gel, and avoiding micromovements that could artifact the exploration, we successfully analyzed its shape (i.e., regular/irregular), the presence of ulceration, its delimitation from the neighboring tissues, the echogenicity, the presence of hyperechoic points inside, and the posterior acoustic shadowing, by employing sonographic techniques.

The tumor thickness was quantified by measurements along two axes (longitudinal and transverse), starting from the granular layer of the epidermis (below the stratum corneum’s hyperechoic band) and extending to the point of maximal tumor infiltration.

The tumor invasion level was classified into five stages. We considered I—superficial to the basement membrane, II—papillary dermis, III—papillary and reticular junction, IV—reticular junction, and V—subcutaneous fat.

Lastly, tumor vascularization was quantified using color Doppler mode, adjusted to the circulation speeds of the skin followed by the exploration of the presence or absence of vascularization, along with the number of vascular pedicles. Consequently, the vascularization was graded as follows: 1—one vascular pedicle, 2—more than one vascular pedicle but no more than three, and 3—more than three vascular pedicles.

### 2.2. Dermoscopic Evaluation

The dermatoscopic evaluation was performed using the Delta 30 dermatoscope and the Vidix 4.0 videodermoscope equipped with the Vectra software (Version 7.4.7). The images were evaluated by two experienced dermatologists, blinded to the histopathological diagnosis and the selection of the following criteria was based on recent data published in the literature [21,22].

For the entire group of patients, we used the following dermatoscopic criteria: the presence of monomorphic/polymorphic vessels, dotted vessels, glomerular vessels, linear vessels, hairpin vessels, serpentine/corkscrew vessels, arborizing vessels, white and yellow scales, white halos surrounding vessels, white and wide follicles, background erythema, rosettes, ulceration/bleeding, erosions, blood spots, white structureless areas, white circles surrounding follicles, presence of pigmentation, and keratin distribution.

### 2.3. Histopathological Evaluation

After clinical and non-invasive imaging evaluation, the skin tumors were excised with oncological safety margins, following the hospital protocol. The tumor tissue was fixed with hematoxylin-eosin and examined under an optical microscope. The histopathological criteria assessed included the type of squamous cell carcinoma (in situ/invasive), degree of differentiation (G1—well differentiated, G2—moderately differentiated, G3—poorly differentiated), tumor thickness, level of tumor invasion, presence of ulceration, keratinization, elastosis, and perineural and angiolymphatic invasion. Additionally, some tumors were analyzed immunohistochemically, but the data were not standardized, and therefore, this analysis was not included.

### 2.4. Ex Vivo Confocal Fluorescence Microscopy

Using FCM, 24 cSCCs were analyzed. Part of the tumor tissue was fixed in Tissue-Tek O.C.T and frozen at −80 degrees Celsius for 30 min. In order to be analyzed under an FCM, the tumors were sectioned with a cryotome with a thickness of 5 microns and applied to microscopy slides (4 sections per slide). The slides were shipped to a confocal microscopy center for analysis in refrigerated containers. Without the use of special staining, the analysis was carried out on native sections.

Fluorescence confocal images of SCCs were captured using a super-resolution Re-Scan Confocal Microscopy system (RCM-VIS unit) acquired from Confocal.nl (Amsterdam, The Netherlands). The system was mounted on a Nikon ECLIPSE Ti2-E inverted microscope with a Plan-Apochromat 10× objective (N.A. = XX). A diode laser set at 488 nm (TOPTICA Photonics AG, Martinsried/Munich, Germany) was specifically used to generate the RCM-Vis images. The data were then analyzed using the NIS Elements software (version 5.11.02).

In order to determine the histologically consecrated characteristics linked in fluorescence confocal microscopy, the sections were examined in collaboration with a physicist who had substantial expertise in confocal microscopy.

The FCM criteria selected were chosen after an extensive review of the literature. We focused on the presence of erosion/ulceration, hyperkeratosis, parakeratosis, architectural disarrangement, plump bright or speckled cells in the epidermis or dermis, nest-like structures in the dermis, keratin pearls, and peritumoral inflammatory infiltrate, the criteria proposed by Hartmann D et al. [23].

### 2.5. Statistical Analysis

The statistical analysis was performed using MedCalc^®^ Statistical Software version 23.1.6 (MedCalc Software Ltd., Ostend, Belgium; https://www.medcalc.org; accessed 22 February 2025). Quantitative variables were characterized by the median and the 25th and 75th percentiles (non-normal distribution). Qualitative variables were expressed by frequency and percentage. Comparisons between groups for quantitative variables were performed using the Mann–Whitney U test, while for qualitative variables, the chi-square test was used. A *p*-value of <0.05 was considered statistically significant.

## 3. Results

The study included 35 cases of histopathologically confirmed cSCC, of which 48.6% were present in men and 51.4% in women. The age had a median of 81 years. The most common tumor location was on the forehead (31.4%), followed by the cheek (28.6%). The predominant Fitzpatrick phototype was type IV (40%). Overall, 54.3% of the cases had a positive history of SCC, while 14.3% presented with an associated field of cancerization. The median clinical maximum diameter was 2 cm. The clinical and demographic characteristics are shown in Table 1.

Regarding the histopathological characteristics, 8 (22.9%) were in situ cSCC, and 27 (77.1%) were invasive cSCC. Differentiation was graded into three stages (G1, G2, and G3). We observed a predominance of G1—27 cases (77.1%), followed by G2 (4, 11.4%) and G3 (4, 11.4%). The histopathologically determined tumor thickness had a median of 2 mm.

### 3.1. Ultrasound

**Figure 1:**
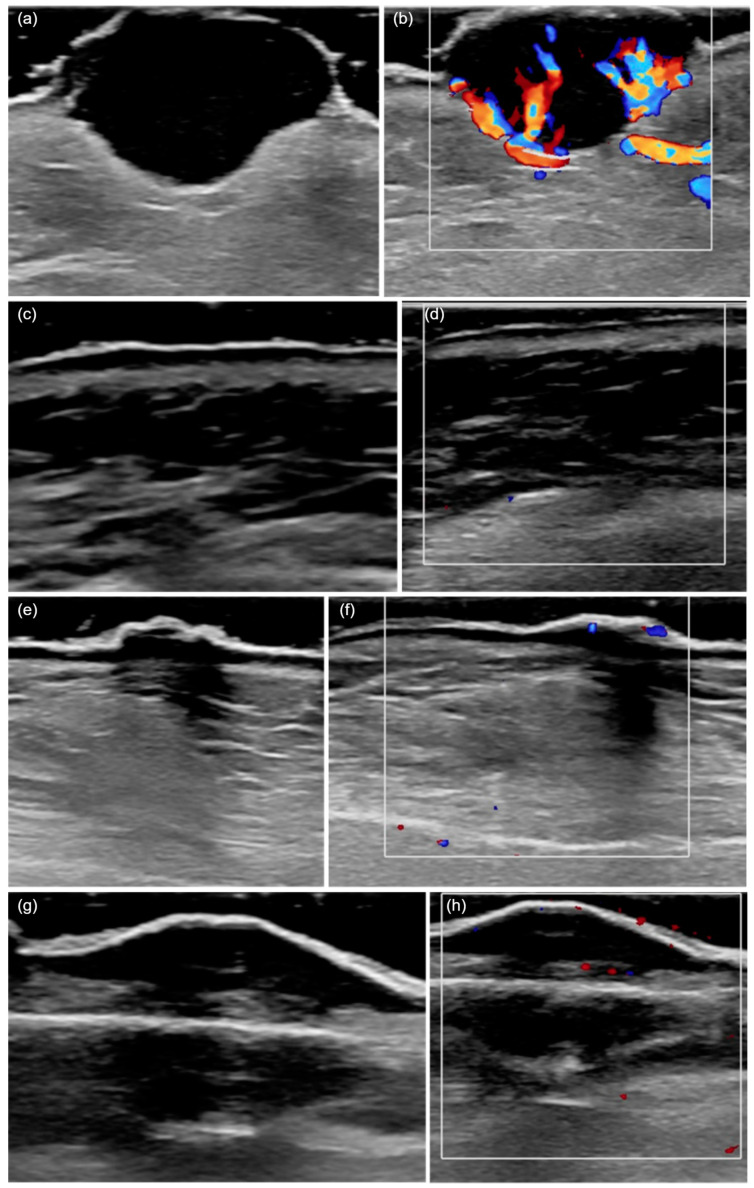
HFUS (20 MHz) features of cutaneous squamous cell carcinoma. (**a**) A hypoechoic lesion with an irregular shape and heterogeneous echogenicity, invading the deep dermis and presenting ulceration. (**b**) The color Doppler mode shows a marked increase in local vascularization. (**c**) A hypoechoic tumor confined to the epidermis, with a regular shape and homogeneous echogenicity. (**d**) An absence of local vascularization in color Doppler mode. (**e**) The presence of posterior acoustic shadowing. (**f**) Moderate vascularization detected in color Doppler mode. (**g**) A hypoechoic, regular, and poorly defined lesion invading the hypodermis, with posterior acoustic shadowing. (**h**) The presence of vascularization in color Doppler mode.

Among the most frequently encountered US criteria, we highlight imprecise boundaries (62.9%), presence of ulceration (60%), hypervascularization (71.4%), and posterior acoustic shadowing (51.4%). The main US characteristics of the tumors are shown in Table 2 and illustrated in Figure 1.

Further, we identified correlations and associations between the different histopathological and ultrasonographic characteristics (Table 3). The presence of vascularization on ultrasound was more frequently associated with invasive cSCC and with histopathologically ulcerated tumors (*p* = 0.027, *p* = 0.006). On the other hand, all eight in situ cSCCs exhibited homogeneous echogenicity (*p* = 0.017).

A strong correlation was found between the tumor thickness determined by US and that determined histopathologically, with Spearman’s rho coefficient r = 0.989 (*p* < 0.001) for the entire patient cohort. An acceptable correlation was observed between the level of tumor invasion determined histopathologically and that determined by US, with Spearman’s rho coefficient r = 0.887 (*p* < 0.001).

From the total number of tumors, 19 of them (90.5%) showed ulceration both on US and histopathologically, with a Kappa correlation coefficient of 0.762 (*p* < 0.001).

The presence of vascularization on US was directly proportional to the tumor thickness determined histopathologically (*p* < 0.001). Tumors that exhibited US vascularization were associated with an average tumor thickness of 3 (1.6; 9) mm. In contrast, tumors without visible vascularization on US were associated with an average tumor thickness of 0.55 (0.1; 1) mm.

The number of vascular pedicles (median of 2) and the presence of ulceration on US were more frequently associated with invasive cSCC (*p* = 0.003, *p* = 0.027). On the other hand, the absence of vascular pedicles and ulceration identified on US was associated with in situ SCC (*p* = 0.003, *p* = 0.0027).

The presence of homogeneous echogenicity on US was associated with smaller tumor thickness (median 1 mm) and a tumor invasion level of II, suggesting an inversely proportional variation. The presence of posterior acoustic shadowing on US varied directly with tumor thickness and the level of tumor invasion (*p* = 0.765, *p* = 0.904).

The presence of vascularization on US was more frequently associated with tumors that exhibited vascular polymorphism on dermatoscopy (*p* = 0.027). Tumors that showed vascular monomorphism on dermatoscopy did not exhibit vascular pedicles on ultrasound (*p* = 0.003).

### 3.2. Dermatoscopy

**Figure 2:**
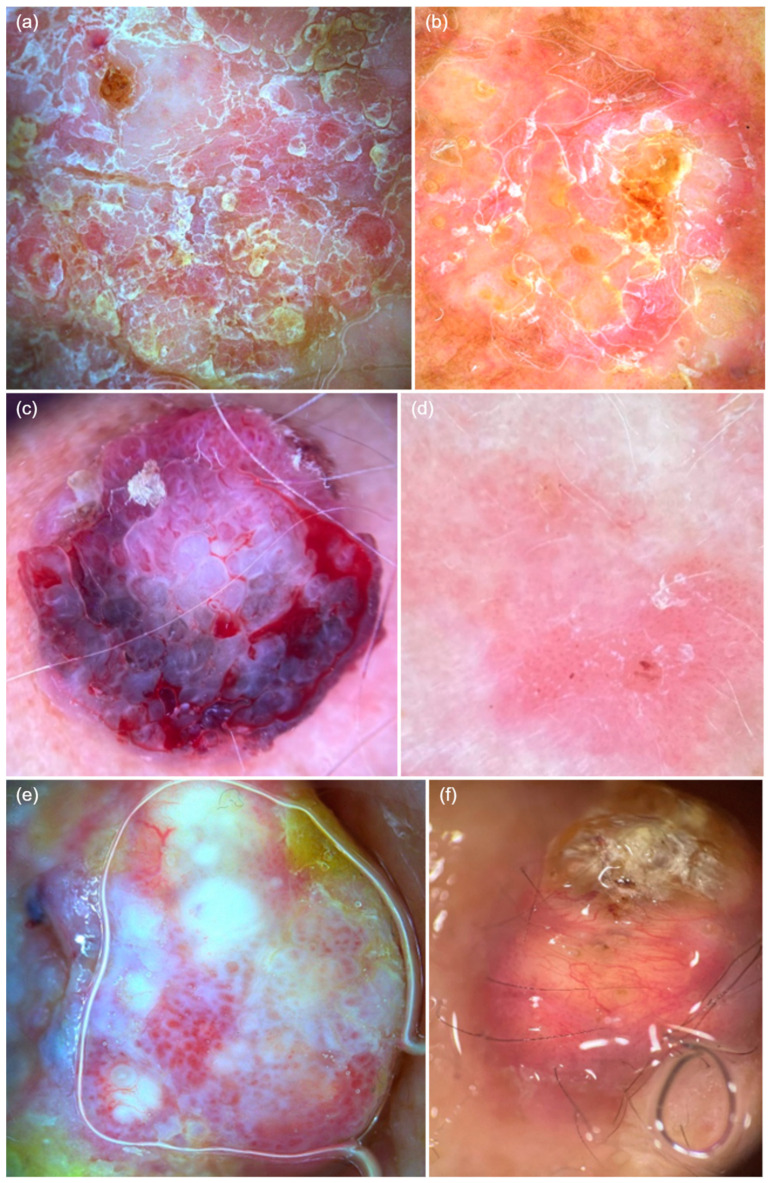
Dermatoscopic features of cutaneous squamous cell carcinoma. (**a**) Glomerular vessels, diffusely distributed mixed scaling, white halos surrounding vessels, erosions, and white structureless areas. (**b**) Punctate vessels on a background erythema, diffuse white scaling, and erosions. (**c**) Polymorphic vessels, including glomerular, serpentine, and linear vessels, along with ulceration, bleeding, and blood spots. (**d**) Punctate vessels on a background erythema with discrete white scaling, white halos surrounding vessels, and white and wide follicles, with central erosion. (**e**) Polymorphic vessels, including glomerular, linear hairpin, and arborizing vessels, along with white structureless areas and white halos surrounding vessels. (**f**) Whitish keratin deposits, polymorphic vessels, including linear hairpin and serpentine vessels.

We analyzed the dermatoscopic diagnostic criteria for cSCC in relation to histopathological characteristics. Based on our observations, the presence of dotted vessels, white scale, white halos surrounding vessels, white and wide follicles, white circles surrounding follicles, a background erythema, and rosettes were more frequently associated with in situ cSCC. Additionally, criteria such as the presence of linear vessels, serpentine/corkscrew vessels, white and yellow scale, ulceration/bleeding, blood spots, and arborizing vessels were more frequently associated with invasive cSCC. The dermatoscopic features are presented in Figure 2. Histopathologically, G1 differentiation was more commonly associated with tumors showing linear vessels or serpentine/corkscrew vessels, white halos surrounding vessels, and a background erythema.

White structureless areas were frequently identified across all three grades of differentiation, while G2 and G3 were more commonly associated with a linear vascular pattern, serpentine/corkscrew vessels, and ulceration/bleeding. On the other hand, the presence of histopathological ulceration was directly proportional to the criteria associated with invasive cSCC and inversely proportional to those associated with in situ cSCC. The main dermatoscopic features in relation to histopathological criteria are highlighted in Table 4.

Dermatoscopically, we analyzed four patterns of keratin distribution. Most G1 and G2 differentiated cSCC cases were associated with diffusely distributed keratin, while G3 differentiated cases did not show keratin deposits on dermatoscopy (*p* = 0.004).

Histopathological keratinization was more frequently associated with the presence of linear vessels (*p* = 0.32), serpentine/corkscrew vessels (*p* = 0.62), white and yellow scale (*p* = 0.003), white structureless areas (*p* = 0.55), and diffuse keratinization on dermatoscopy (*p* = 0.001).

We observed that tumor thickness varied directly proportionally to criteria such as the presence of linear vessels, hairpin vessels, serpentine/corkscrew vessels, ulceration/bleeding, and was inversely proportional to the presence of dotted vessels, white and wide follicles, a background erythema, and erosions (Table 5).

### 3.3. Ex Vivo Confocal Fluorescence Microscopy

A total of 24 cases were analyzed using FCM according to the previously detailed technique. Among these, 5 were in situ cSCC and 19 were invasive cSCC. The most representative grade of differentiation was G1 (19 cases), followed by G2 (3 cases) and G3 (1 case).

**Figure 3:**
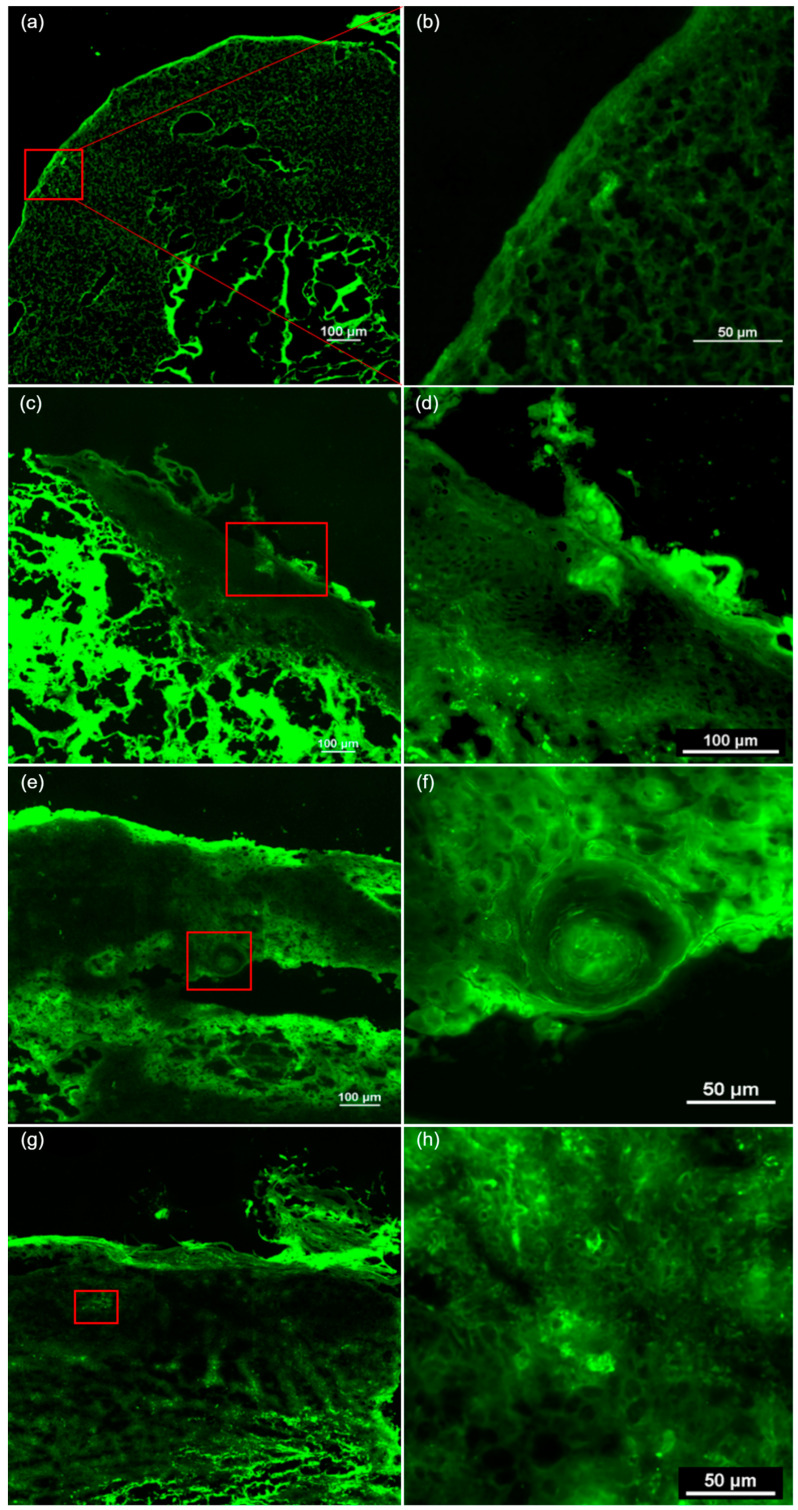
The ex vivo confocal fluorescence microscopy features of cutaneous squamous cell carcinoma. (**a**) A tumor presenting ulceration, hyperkeratosis, architectural disarrangement, and plump bright or speckled cells in both the epidermis and dermis. (**b**) The presence of hyperkeratosis and parakeratosis in the stratum corneum of the epidermis. (**c**) A tumor invading the dermis, showing hyperkeratosis, architectural disarrangement, plump bright or speckled cells in the epidermis and dermis, and nest-like structures in the dermis. (**d**) Hyperkeratosis and parakeratosis in the stratum corneum. (**e**) A tumor invading the hypodermis, with architectural disarrangement, plump bright or speckled cells in the epidermis and dermis. (**f**) The presence of keratin pearls and nest-like structures in the dermis. (**g**) Hyperkeratosis, parakeratosis, architectural disarrangement, and plump bright or speckled cells in the epidermis and dermis. (**h**) Plump bright or speckled cells arranged in nests in the epidermis.

**Figure 4:**
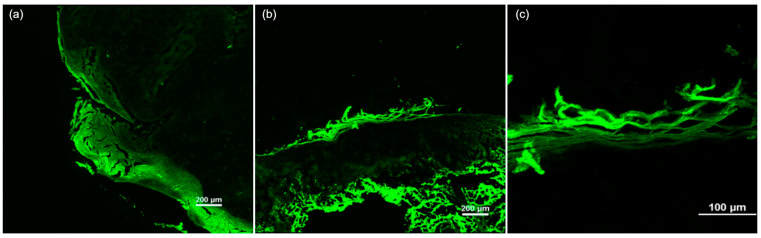
The ex vivo confocal fluorescence microscopy features of cutaneous squamous cell carcinoma. (**a**) A tumor invading both the epidermis and dermis, showing architectural disarrangement and hyperkeratosis. (**b**) A squamous cell carcinoma in situ presenting hyperkeratosis, architectural disarrangement, and plump bright or speckled cells in the epidermis, with an absence of these features in the dermis. (**c**) Hyperkeratosis with parakeratosis in the stratum corneum.

Regarding the FCM criteria, in situ cSCC was more frequently associated with the presence of hyperkeratosis, architectural disarrangement, and plump bright or speckled cells in the epidermis. In these cases, no plump bright or speckled cells were identified in the dermis, nor were nest-like structures in the dermis or keratin pearls observed. Invasive cSCC was more frequently associated with the presence of erosion/ulceration, hyperkeratosis, parakeratosis, architectural disarrangement, and plump bright or speckled cells in both the epidermis and dermis. The ex vivo FCM features of cSCC are presented in Figure 3 and Figure 4.

Regarding the histopathological degree of differentiation, the FCM criteria were encountered in all cases. In G1 cSCC, the most frequent criteria were hyperkeratosis, parakeratosis, architectural disarrangement, and plump bright or speckled cells in the epidermis. In G3 cSCC, the presence of hyperkeratosis and keratin pearls was not observed (Table 6).

The presence of FCM criteria such as architectural disarrangement or plump bright or speckled cells in the epidermis did not vary depending on tumor thickness and level of invasion. In contrast, the presence of erosion/ulceration and parakeratosis was significantly associated with a median tumor thickness of ≤2 mm and a median invasion level of ≤III.

The presence of plump bright or speckled cells in the dermis and nest-like structures in the dermis was significantly associated (*p* = 0.002) with thicker tumors (median = 3 mm) and deeper invasion (median = IV).

## 4. Discussion

Using specific dermatoscopic patterns allows for the differentiation of cSCC from other nonmelanocytic tumors and enhances the detection of early invasive progression in precursor lesions. Skin US proves invaluable in assessing tumor size, vascularity, depth, and proximity to nearby structures. In vivo RCM offers high-resolution, non-invasive imaging that distinguishes invasive cSCC from in situ cases, while ex vivo RCM provides an efficient method for identifying residual tumor tissue, complementing Mohs surgery. LC-OCT delivers real-time dual-orientation imaging, improving cSCC subtype classification with deeper tissue penetration.

These novel methods offer speed, non-invasiveness, and enhanced diagnostic sensitivity, yet their broader adoption is limited by high costs, the need for specialized expertise, and anatomical constraints. As technology improves, these techniques will likely become more widely used, contributing to better skin cancer diagnosis and treatment [13].

Our findings reflect cSCC as a disease predominantly affecting elderly patients, with a balanced gender distribution and frequent occurrence on sun-exposed areas, reinforcing the role of chronic UV exposure. The predominance of well-differentiated and moderately thick tumors suggests a generally favorable prognosis in this cohort. The higher proportion of invasive over in situ SCC highlights the need for improved early recognition, especially in high-risk anatomical zones.

On US imaging, cSCC typically appears as a heterogeneously hypoechoic lesion with irregular borders, absent hyperechoic spots, and a tendency to involve the deeper layers. The vascular pattern is diffusely increased throughout the mass, unlike BCC, where neovascularity is less prominent and usually found at the lesion’s base. However, due to the effect of hyperkeratosis, many lesions may show limited blood flow signals, which do not fully represent the actual vascularity [24,25].

Zhu et al. [26] evaluated HFUS features for diagnosing cSCC and its precursors, actinic keratosis (AK) and Bowen’s disease (BD). The authors found that HFUS is valuable for differentiating between AK, BD, and invasive cSCC, suggesting its potential for dynamic and non-invasive monitoring in the spectrum of cSCC.

Among the most commonly observed US features, our study highlights imprecise tumor boundaries (62.9%), ulceration (60%), hypervascularization (71.4%), and posterior acoustic shadowing (51.4%).

In the study conducted by Chen et al. [14] on 68 SCC cases with a median thickness of 5.9 mm, the findings were as follows: irregular lesion shape in 61.8%, convex lesion surface margin in 52.9%, irregular lesion base margin in 41.2%, homogeneous echogenicity in 69%, posterior acoustic shadowing in 66.2%, absence of hyperechoic spots in 76.5%, dermal layer involvement in 64.1%, and a Doppler vascularity pattern of III (dense linear blood flow signals with unclear distribution) in 66.2% of cases.

Vascularization detected on US was more frequently associated with invasive SCC and histopathologically confirmed ulcerated tumors (*p* = 0.027, *p* = 0.006). Valian et al. showed that 95% of patients with SCC (20 cases) had positive tumor vascularity but found no significant correlation between disease type and vascularity (*p* = 0.6), indicating that hypervascularity alone is insufficient for differentiating cSCC subtypes [27].

Ulceration was identified in 90.5% of tumors on both US and histopathology, with a Kappa correlation coefficient of 0.762 (*p* < 0.001). Additionally, US vascularization was directly proportional to tumor thickness as determined histopathologically (*p* = 0.007). Tumors exhibiting vascularization on US had an average thickness of 3 (1.6; 9) mm, whereas those without visible vascularization measured an average of 0.5 (0.1; 1).

A higher number of vascular pedicles (median of 2) and the presence of ulceration on US were more frequently linked to invasive SCC (*p* = 0.003, *p* = 0.027). Conversely, in situ SCC was more commonly associated with the absence of vascular pedicles and ulceration on US (*p* = 0.003, *p* = 0.0027).

Furthermore, vascularization on US was more frequently observed in tumors exhibiting vascular polymorphism on dermatoscopy (*p* = 0.027). Tumors displaying vascular monomorphism on dermatoscopy did not show vascular pedicles on US (*p* = 0.003).

Chen et al. [14] showed that on dermoscopy, cSCC typically presents with numerous curved hairpin, linear-irregular, or glomerular blood vessels. Similarly, HFUS reveals dense linear blood flow signals with an uneven distribution over approximately half of the lesion. However, posterior acoustic shadowing beneath the keratinized areas can sometimes obscure the visibility of blood flow, particularly in cSCC cases.

A strong correlation was found between tumor thickness measured by US and that determined histopathologically, with Spearman’s rho coefficient of r = 0.989 (*p* < 0.001) across the entire patient cohort. A similarly high correlation was observed between the level of tumor invasion assessed histopathologically and by US (r = 0.887, *p* < 0.001). In their study, Valian et al. [27] similarly found a high correlation coefficient (0.912) between ultrasound and pathology for tumor size and depth. A study by Bergón-Sendín M et al., involving 40 patients with cSCC who underwent Methotrexate treatment, found a positive correlation between ultrasound measurements and histological evaluation of tumor diameter and thickness (r = 0.932, *p* < 0.01) [28].

Homogeneous echogenicity on US was correlated with smaller tumor thickness (median: 1 mm) and a tumor invasion level of II, indicating an inversely proportional relationship. All eight cases of in situ cSCC displayed homogeneous echogenicity (*p* = 0.017).

Posterior acoustic shadowing on US correlated directly with tumor thickness and invasion level (*p* = 0.765, *p* = 0.904).

Prior studies report dermoscopy sensitivity of 79% and specificity of 87% for cSCC detection. Bowen’s disease (BD) has a higher sensitivity of 98%, making dermoscopy a highly reliable diagnostic tool for BD [29].

Lallas et al. [30] observed notable differences in the dermoscopic patterns of poorly differentiated SCC compared to well- and moderately differentiated tumors. White-colored features, such as keratin, white circles, and structureless whitish areas, were commonly associated with well- and moderately differentiated variants. A central distribution of keratin emerged as a strong indicator of well-differentiated tumors. In contrast, poorly differentiated SCC predominantly displayed a red hue due to dense vascularization, lacking keratin or other white-colored characteristics.

Zalaudek et al. [31] reported similar dermoscopic characteristics for SCC. Well-differentiated cSCC (Keratoacanthoma-type) is marked by a central keratin mass, encircled by elongated telangiectasias, or as a polymorphic vascular pattern, including hairpin and dotted vessels.

Moderately differentiated cSCC commonly presents with peripheral hairpin vessels and diffuse, structureless yellow-to-light brown or white keratotic areas, often accompanied by extensive and variable ulceration. Additional distinguishing features include targetoid-appearing hair follicles (white circles), diffuse white structureless areas, keratin masses interspersed with blood spots, and ulceration. Poorly differentiated SCC subtypes generally lack keratinization. Instead, they display polymorphous vascular patterns consisting of small-caliber linear vessels, hairpin vessels, or glomerular vessels set against a reddish background. In certain cases, peripheral structureless white areas may be present, providing a key diagnostic indicator [31].

We evaluated the dermatoscopic diagnostic criteria for cSCC in relation to histopathological features. In situ cSCC was more frequently associated with dotted vessels, white scale, white halos around vessels, white and wide follicles, white circles surrounding follicles, a background erythema, and rosettes. In contrast, invasive SCC was more commonly linked to linear vessels, serpentine/corkscrew vessels, white and yellow scale, ulceration/bleeding, blood spots, and arborizing vessels.

Histopathologically, G1 differentiation was primarily observed in tumors with linear or serpentine/corkscrew vessels, white halos around vessels, and a background erythema. G2 and G3 differentiation were more often associated with a linear vascular pattern, serpentine/corkscrew vessels, and ulceration/bleeding. Notably, histopathological ulceration showed a direct correlation with invasive SCC criteria and an inverse correlation with in situ SCC characteristics.

Our analysis of keratin distribution revealed that most G1 and G2 SCC cases exhibited diffuse keratin deposits, whereas G3 cases lacked keratin on dermatoscopy (*p* = 0.004).

Additionally, tumor thickness showed a direct correlation with the presence of linear vessels, hairpin vessels, serpentine/corkscrew vessels, and ulceration/bleeding. Conversely, it was inversely related to features such as dotted vessels, white and wide follicles, a background erythema and erosions.

The selection of ex vivo FCM criteria was based on a comprehensive review of the literature. Hartmann D et al. proposed the following parameters for the diagnosis of cSCC: erosion/ulceration, hyperkeratosis, parakeratosis, and architectural disarrangement, plump bright or speckled cells in the epidermis or dermis, nest-like structures in the dermis, keratin pearls, and peritumoral inflammatory infiltrate [23]. Longo et al. [32] evaluated the use of ex vivo FCM during Mohs surgery for cSCC. The study found that integrating FCM allowed for the rapid assessment of excision margins, potentially improving surgical outcomes by ensuring complete tumor removal.

In our study, in situ cSCC was more commonly associated with hyperkeratosis, architectural disarrangement, and plump bright or speckled cells in the epidermis. In contrast, invasive cSCC was frequently linked to erosion/ulceration, hyperkeratosis, parakeratosis, architectural disarrangement, and the presence of plump bright or speckled cells in both the epidermis and dermis.

When analyzing the histopathological differentiation grade, FCM criteria were present in all cases. In G1 cSCC, the most commonly observed features included hyperkeratosis, parakeratosis, architectural disarrangement, and plump bright or speckled cells in the epidermis. However, in G3 cSCC, neither hyperkeratosis nor keratin pearls were identified.

Despite some promising research, such as studies focusing on specific imaging features or combining FCM with Mohs surgery, the broader application of ex vivo FCM for cSCC diagnosis and management remains underexplored. Most existing studies on FCM in cSCC are relatively small-scale or focused on in vivo applications [33]. While ex vivo FCM has been explored for tumor margin evaluation, the technique has not been extensively studied in large, well-controlled cohorts to establish its routine clinical utility.

There is a gap in research that directly assesses the sensitivity, specificity, and diagnostic accuracy of ex vivo FCM when compared to these traditional techniques. The adoption of FCM in clinical practice requires high technical expertise and specialized equipment, which may limit its widespread use and, consequently, the number of studies published in the field.

Non-invasive diagnostic methods can aid in determining tumor depth and differentiation grade, which may influence treatment decisions. In certain cases, particularly in situ SCC located in critical areas, less invasive treatments such as photodynamic therapy or topical chemotherapy may be preferred over surgical excision. Additionally, correlating US and histopathological findings with established tumor staging systems (TNM, BWH) could help identify specific characteristics associated with poorer prognosis. This approach may facilitate early identification of aggressive tumors, guiding clinicians toward a more tailored therapeutic strategy that balances oncologic safety with functional and esthetic considerations.

Artificial intelligence (AI) could play a valuable role in this field by enhancing the interpretation of ultrasound and dermatoscopic images for cSCC. AI-driven algorithms have already demonstrated high accuracy in detecting and classifying skin lesions, and their application to ultrasonographic features of cSCC could improve diagnostic precision, particularly in distinguishing in situ from invasive lesions. Furthermore, AI could assist in automated tumor staging by correlating imaging findings with histopathological characteristics and established classification systems. Future research should explore AI’s potential in refining non-invasive diagnostic techniques, predicting tumor aggressiveness, and personalizing treatment strategies, thereby optimizing patient outcomes while reducing unnecessary surgical interventions.

There are several limitations in this study. The study involved a relatively small sample size of 35 cSCC cases, which limits the generalizability of the findings. A large number of associations were tested within a small sample size, increasing the likelihood that some findings may be statistically significant but not clinically meaningful. A larger cohort could provide a more robust understanding of the correlations between imaging diagnostic techniques and histopathological characteristics. The research was conducted at a single center, which may limit the external validity of the results. Differences in patient populations, diagnostic practices, and technology available at other institutions may affect the generalizability of the findings. The study did not incorporate long-term follow-up to assess the clinical outcomes of the patients based on the diagnostic criteria and imaging techniques used. Longitudinal data would be valuable to understand the predictive value of the imaging methods for recurrence or metastasis. Different operators may have varying levels of expertise in using the diagnostic imaging tools. This variability could influence the consistency and reliability of imaging results, particularly in more complex cases.

Another limitation of our study is the lack of a systematic, case-by-case correlation of specific features across all diagnostic modalities, which could have provided deeper insights and enhanced the robustness of our findings.

## 5. Conclusions

The uniqueness of this study lies in its comprehensive assessment of advanced imaging techniques for diagnosing cSCC, combining dermatoscopy, skin ultrasound, and ex vivo FCM. While previous studies have examined these modalities separately, this research uniquely integrates and correlates their findings with histopathological features, providing novel insights into the associations between imaging characteristics, tumor grade, vascularity, and invasion depth. Additionally, the study highlights the value of non-invasive imaging techniques such as RCM and LC-OCT, which offer high-resolution, real-time visualization to support more precise treatment planning. By integrating imaging data with clinical and histopathological evaluations, this research makes a significant contribution to the evolving landscape of non-melanoma skin cancer diagnosis.

## Figures and Tables

**Table 1:** Clinical and demographic characteristics.

Variable		Characteristics
Anatomical location (%)	Forearm	3 (8.6%)
	Arm	1 (2.9%)
	Lip	1 (2.9%)
	Thigh	2 (5.7%)
	Finger	1 (2.9%)
	Forehead	11 (31.4%)
	Leg	1 (2.9%)
	Nose	1 (2.9%)
	Cheek	10 (28.6%)
	Thorax	3 (8.6%)
	Ear	1 (2.9%)
	Total	35 (100%)
Sex (%)	Male	17 (48.6%)
	Female	18 (51.4%)
Border delimitation (%)	Imprecise	19 (54.3%)
	Precise	16 (45.7%)
Phototype (%)	II	10 (28.6%)
	III	11 (31.4%)
	IV	14 (40%)
History of cSCC (%)	Yes	19 (54.3%)
	No	16 (45.7%)
Ulceration (%)	Yes	28 (80%)
	No	7 (20%)
Keratin on the surface (%)	Yes	31 (88.6%)
	No	4 (11.4%)
Cancerization field (%)	Yes	5 (14.3%)
	No	30 (85.7%)
Age (years)	Median (25; 75 percentile)	81 (78; 85)
Clinical diameter (cm)	Median (25; 75 percentile)	2 (0.2; 4)

**Table 2:** Ultrasound characteristics of the tumors.

Variable		Characteristics
Shape (%)	Regular	20 (57.1%)
	Irregular	15 (42.9%)
Level of invasion (%)	I	6 (17.1%)
	II	4 (11.4%)
	III	5 (14.3%)
	IV	10 (28.6%)
	V	10 (28.6%)
Delimitation (%)	Imprecise	22 (62.9%)
	Precise	13 (37.1%)
Ulceration (%)	Present	21 (60%)
Vascularization (%)	Present	25 (71.4%)
Homogeneous echogenicity (%)	Present	20 (57.1%)
Hyperechoic points (%)	Present	6 (17.1%)
Posterior acoustic shadowing (%)	Present	18 (51.4%)
Tumor thickness (mm)	Median (25; 75 percentile)	2.2 (0.5; 5)

**Table 3:** The distribution of ultrasonographic criteria based on histopathological characteristics.

Variable		US Characteristics							
		Vascularization (%)	*p*	Homogeneous Echogenicity (%)	*p*	Hyperechoic Points (%)	*p*	Posterior Acoustic Shadowing (%)	*p*
		Present	**0.027**	Present	**0.017**	Present	1.00	Present	0.443
cSCC stage (%)	In situ	3 (12%)		8 (40%)		1 (16.7%)		3 (16.7%)	
	Invasive	22 (88%)		12 (60%)		5 (83.3%)		15 (83.3%)	
Degree of differentiation (%)	G1	18 (72%)	0.382	17 (85%)	0.342	4 (66.7%)	0.798	15 (83.3%)	0.521
	G2	3 (12%)		2 (10%)		1 (16.7%)		1 (5.6%)	
	G3	4 (16%)		1 (5%)		1 (16.7%)		2 (11.1%)	
Ulceration (%)	Present	19 (76%)	**0.006**	10 (50%)	0.296	3 (50%)	0.664	13 (72.2%)	0.241
	Absent	6 (24%)		10 (50%)		3 (50%)		5 (27.8%)	
Keratinization (%)	Present	21 (84%)	1.00	18 (90%)	0.631	5 (83.3%)	1.00	16 (88.9%)	0.658
	Absent	4 (16%)		2 (10%)		1 (16.7%)		2 (11.1%)	

**Table 4:** Dermatoscopic features in relation to histopathological criteria.

Variable		Histopathological Characteristic								
Dotted vessels (%)		CSS stage (%)		*p*	Degree of differentiation (%)			*p*	Ulceration (%)	*p*
		In situ	Invasive	**<0.001**	G1	G2	G3	0.16		0.43
		7 (77.8%)	2 (22.2%)		9 (100%)	0 (0%)	0 (0%)		4 (44%)	
Glomerular vessels (%)		1 (6.7%)	14 (93.3%)	0.10	12 (80%)	2 (13.3%)	1 (6.7%)	0.72	9 (60%)	1.00
Linear vessels (%)		0 (0%)	21 (100%)	**0.00**	13 (61.9%)	4 (19%)	4 (19%)	**0.03**	15 (71.4%)	0.15
Hairpin vessels (%)		1 (9.1%)	10 (90.9%)	0.38	9 (81.8%)	0 (0%)	2 (18.2%)	0.28	5 (45.5%)	0.28
Serpentine/corkscrew vessels (%)		0 (0%)	21 (100%)	**<0.001**	13 (61.9%)	4 (19%)	4 (19%)	**0.03**	15 (71.4)	0.15
Scale (%)	white	3 (75%)	1 (25%)	**0.02**	4 (100%)	0 (0%)	0 (0%)	**0.003**	0 (0%)	0.004
	yellow	3 (33.3%)	6 (66.7%)		7 (77.8%)	2 (22.2%)	0 (0%)		3 (33.3%)	
	both	2 (13.3%)	13 (86.7%)		13 (86.7%)	2 (13.3%)	0 (0%)		13 (86.7%)	
	no	0 (0%)	7 (100%)		3 (42.9%)	0 (0%)	4 (57.1%)		5 (71.4%)	
White halos surrounding vessels (%)		5 (33.3%)	10 (66.7%)	0.41	14 (93.3%)	1 (6.7%)	0 (0%)	0.16	9 (60%)	1.00
White and wide follicles (%)		6 (46.2%)	7 (53.8%)	**0.03**	12 (92.3%)	1 (7.7%)	0 (0%)	0.27	5 (38.5%)	0.038
Background erythema (%)		8 (53.3%)	7 (46.7%)	**<0.001**	14 (93.3%)	1 (6.7%)	0 (0%)	0.16	7 (46.7%)	0.16
Rosettes (%)		5 (38.5%)	8 (61.5%)	0.21	13 (100%)	0 (0%)	0 (0%)	0.06	7 (53.8%)	0.49
Ulceration/bleeding (%)		0 (0%)	18 (100%)	**0.001**	12 (66.7%)	3 (16.7%)	3 (16.7%)	0.12	16 (88.9%)	0.001
Erosions (%)		4 (50%)	4 (50%)	0.06	8 (100%)	0 (0%)	0 (0%)	0.25	5 (62.5%)	1.00
Blood spots (%)		0 (0%)	12 (100%)	**0.03**	7 (58.3%)	2 (16.7%)	3 (25%)	**0.03**	11 (91.7%)	0.01
White structureless areas (%)		4 (14.8%)	23 (85.2%)	**0.03**	20 (74.1%)	4 (14.8%)	3 (11.1%)	0.31	19 (70.4%)	0.079
White circles surrounding follicles (%)		6 (46.2%)	7 (53.8%)	**0.03**	12 (92.3%)	1 (7.7%)	0 (0%)	0.27	5 (38.5%)	0.038
Presence of pigmentation (%)		1 (100%)	0 (0%)	0.23	1 (100%)	0 (0%)	0 (0%)	0.87	0 (0%)	0.38
Arborizing vessels (%)		0 (0%)	10 (100%)	0.07	6 (60%)	1 (10%)	3 (30%)	**0.01**	7 (70%)	0.70

**Table 5:** The frequency of dermatoscopic criteria depending on the tumor thickness and the level of histopathological invasion.

Variable			Tumor Thickness (mm)		Level of Invasion	
			Median (25; 75 Percentile)	*p*	Median (25; 75 Percentile)	*p*
Dotted vessels			0.00 (0; 0.1)	**<0.001**	I (I; I)	**<0.001**
Glomerular vessels			1.8 (1; 2.7)	0.52	IV (III; IV)	0.94
Linear vessels			3 (2; 8.4)	**<0.001**	IV (IV; V)	**<0.001**
Hairpin vessels			1.25 (1; 3.25)	0.80	III (II; IV)	0.46
Serpentine/corkscrew vessels			3.2 (2; 8.9)	**<0.001**	IV (IV; V)	**<0.001**
Scale	White		0.00 (0; 0.7)	**0.02**	I (I; III)	0.19
	Yellow		2 (0; 4.8)		IV (I; IV)	
	Both		2.7 (1; 3.5)		III (III; IV)	
	No		6.7 (0.8; 14.7)		IV (II; V)	
White halos surrounding vessels			1.1 (0; 2.2)	**0.02**	III (I; IV)	**0.04**
White and wide follicles			0.2 (0; 1.2)	**0.002**	I (I; III)	**0.007**
Background erythema			0.00 (0; 1.2)	**<0.001**	I (I; III)	**0.001**
Rosettes			1 (0; 2.8)	0.07	III (I; III)	**0.03**
Ulceration/bleeding			3.7 (2; 9.1)	**<0.001**	IV (IV; V)	**<0.001**
Erosions			0.1 (0; 1)	**0.002**	I (I; III)	**0.002**
Blood spots			5.5 (3.1; 9.8)	**<0.001**	IV (IV; V)	**0.001**
White structureless areas			2.2 (1; 4)	0.068	IV (III; V)	**0.008**
White circles surrounding follicles			0.2 (0; 1.2)	**0.002**	I (I; III)	**0.007**
Arborizing vessels			6.4 (3;11)	**0.001**	IV (IV; V)	**<0.001**
Keratin distribution		Absent	6.7 (0.8; 14)	0.12	IV (II; V)	0.42
		Central	2.7 (0; 5.2)		IV (I; V)	
		Diffuse	1.6 (0.2; 3)		III (I; IV)	
		Peripheral	1 (0; 1)		IV (I; IV)	

**Table 6:** The frequency of FCM diagnostic criteria related to histopathological characteristics.

Variable	Histopathological Characteristic								
	CSS Stage (%)		*p*	Degree of Differentiation (%)			*p*	Ulceration (%)	*p*
	In Situ	Invasive		G1	G2	G3			
Erosion/ulceration (%)	2 (40%)	17 (89.5%)	**0.04**	15 (75%)	3 (100%)	1 (100%)	0.53	17 (100%)	**<0.001**
Hyperkeratosis (%)	5 (100%)	18 (94.7%)	1.00	20 (100%)	3 (100%)	0 (0%)	**<0.001**	17 (100%)	0.29
Parakeratosis (%)	4 (80%)	18 (94.7%)	0.38	19 (95%)	3 (100%)	0 (0%)	**0.003**	17 (100%)	0.07
Architectural disarrangement (%)	5 (100%)	19 (100%)	-	20 (100%)	3 (100%)	1 (100%)	-	17 (100%)	-
Plump bright or speckled cells in the epidermis (%)	4 (80%)	19 (100%)	0.20	19 (95%)	3 (100%)	1 (100%)	0.90	17 (100%)	0.29
Plump bright or speckled cells in the dermis (%)	0 (0%)	16 (84.2%)	**0.001**	12 (60%)	3 (100%)	1 (100%)	0.30	14 (82.4%)	**0.02**
Nest-like structures in the dermis (%)	0 (0%)	16 (84.2%)	**0.001**	12 (60%)	3 (100%)	1 (100%)	0.30	14 (82.4%)	**0.02**
Keratin pearls (%)	0 (0%)	7 (36.8%)	0.27	6 (30%)	1 (33.3%)	0 (0%)	0.80	5 (29.4%)	1.0

## Data Availability

The original contributions presented in the study are included in the article; further inquiries can be directed to the corresponding author.

## Abbreviations

The following abbreviations are used in this manuscript:

FCM	confocal fluorescence microscopy
BCC	basal cell carcinoma
HFUS	high-frequency ultrasound
cSCC	cutaneous squamous cell carcinoma
lacSCC	locally advanced squamous cell carcinoma
mcSCC	metastatic squamous cell carcinoma
AK	actinic keratoses
RCM	reflectance confocal microscopy
LC-OCT	line-field confocal optical coherence tomography (LC-OCT)
OCT	optical coherence tomography
BD	Bowen’s disease
